# Substance use, risk behaviours and well-being after admission to a quasi-residential abstinence-based rehabilitation programme: 4-year follow-up

**DOI:** 10.1192/bjo.2023.23

**Published:** 2023-03-13

**Authors:** Nina MacKenzie, Daniel J. Smith, Stephen M. Lawrie, Andrew M. Rome, David McCartney

**Affiliations:** Division of Psychiatry, Centre for Clinical Brain Sciences, University of Edinburgh, Edinburgh, UK; and NHS Lothian, Edinburgh, UK; Division of Psychiatry, Centre for Clinical Brain Sciences, University of Edinburgh, Edinburgh, UK; Figure 8 Consultancy Services Ltd, Dundee, UK; Lothian and Edinburgh Abstinence Programme, NHS Lothian, Edinburgh, UK

**Keywords:** Drugs of dependence disorders, rehabilitation, outcome studies, alcohol disorders, opiate disorders

## Abstract

**Background:**

Tackling Scotland's drug-related deaths and improving outcomes from substance misuse treatments, including residential rehabilitation, is a national priority.

**Aims:**

To analyse and report outcomes up to 4 years after attendance at a substance misuse residential rehabilitation programme (Lothians and Edinburgh Abstinence Programme).

**Method:**

In total, 145 participants were recruited to this longitudinal quantitative cohort study of an abstinence-based residential rehabilitation programme based on the therapeutic community model; 87 of these participants were followed up at 4 years. Outcomes are reported for seven subsections of the Addiction Severity Index-X (ASI-X), together with frequency of alcohol use, heroin use, injecting drug use and rates of abstinence from substances of misuse.

**Results:**

Significant improvement in most outcomes at 4 years compared with admission scores were found. Completing the programme was associated with greater rates of abstinence, reduced alcohol use and improvements in alcohol status score (Mann–Whitney *U* = 626, *P* = 0.013), work satisfaction score (*U* = 596, *P* = 0.016) and psychiatric status score (*U* = 562, *P* = 0.007) on the ASI-X, in comparison with non-completion. Abstinence rates improved from 12% at baseline to 48% at 4 years, with the rate for those completing the programme increasing from 14.5% to 60.7% (χ^2^(2, 87) = 9.738, *P* = 0.002). Remaining abstinent from substances at follow-up was associated with better outcomes in the medical (*U* = 540, *P* < 0.001), psychiatric (*U* = 273.5, *P* < 0.001) and alcohol (*U* = 322.5, *P* < 0.001) subsections of the ASI-X.

**Conclusions:**

Attending this abstinence-based rehabilitation programme was associated with positive changes in psychological and social well-being and harm reduction from substance use at 4-year follow-up, with stability of change from years 1 to 4.

Higher-risk alcohol use and problematic drug use are significant issues in Scotland, causing damage to people's lives, families and communities. Tackling the high level of drug-related deaths in Scotland is a priority of the Scottish Government, which recently set out a National Mission to reduce drug deaths through improvements to treatment, recovery and other support services.^1^ This includes increasing capacity and improving access to residential rehabilitation. A review of existing literature conducted on behalf of the Scottish Government found a ‘relatively robust body of evidence suggesting that residential rehabilitation is associated with improvements across a variety of outcomes relating to substance use, health and quality of life’.^2^

Rome et al (2017) reported 6-month and 1-year outcomes following attendance at a quasi-residential rehabilitation programme, and found significant positive changes in participants’ physical health and social well-being and significant harm reduction in relation to alcohol and heroin use.^3^ The long-term impact of attendance at residential rehabilitation programmes is less frequently reported in the research literature but is an important aspect of examining the various stages of addiction and recovery. This paper specifically considers whether improvements in outcomes of the same cohort are maintained or otherwise at 4-year follow-up. Outcomes are reported for seven subsections of the Addiction Severity Index-X (ASI-X) interview tool, together with frequency of alcohol and heroin use, frequency of injecting drug use and rates of abstinence from substances of misuse.

## Aims

To analyse and report outcomes up to 4 years after attendance at a residential substance misuse rehabilitation programme (the Lothians and Edinburgh Abstinence Programme).

## Method

### Setting

The Lothians and Edinburgh Abstinence Programme (LEAP) is a mixed-gender 12-week quasi-residential (treatment and accommodation are on different sites) drug and alcohol rehabilitation programme based on a therapeutic community model. Patients are housed in nearby hostel accommodation provided by the City of Edinburgh Council solely for LEAP patients and they attend the treatment facility for morning and afternoon sessions. The aim of LEAP is to make a positive difference to illicit drug use and harmful alcohol use and to positively influence various social indicators, including crime, training and employment, and social functioning. It is a 3-month-long day programme consisting of psychosocial interventions delivered by a team of NHS Lothian staff. The team includes medical staff (a general practitioner (GP) with special interest and a consultant psychiatrist), a pharmacist, clinical psychologist, therapists, registered mental health nurses, an occupational therapist, a peer support coordinator, peer support workers and administration support staff. Referrals are accepted from community mental health teams (CMHTs), GPs, third-sector community addiction support hubs (which work in collaboration with CMHTs), specialist in-patient detoxification services and criminal justice workers. Similar to other residential rehabilitation facilities, the programme consists of groups, meetings, duties, teaching and a focus on relationships and social roles within the community. There are one-to-one sessions with a therapist and group therapy, using a blend of therapeutic techniques, including cognitive–behavioural therapy. The community of patients and peer support workers act as an agent of change.^4^ Further additions to the LEAP model include physical and mental health assessment and care, housing and benefits support, family support, education, training and employability provision, aftercare for up to 2 years and assertive linkage to mutual aid before and during treatment. All patients are local to the Lothian area, which means that new social networks developed during treatment can continue to be accessed after treatment is concluded.^3^

### Design

The study design was a longitudinal quantitative cohort study. This was chosen to allow the collection of data from a known cohort that had received the same intervention at set intervals over an extended period. The LEAP cohort included every patient admitted to the facility between April 2008 and March 2009. Participants had to be over 18 years of age, commencing a new treatment episode, prepared to give written consent to the tracking/follow-up procedures and willing to provide locator information. Severity of Dependence Scale^5^ measures were used to ascertain the extent to which the baseline measures of the cohort were comparable in severity to other treatment-seeking populations in Scotland.

### Sample

**Fig. 1:**
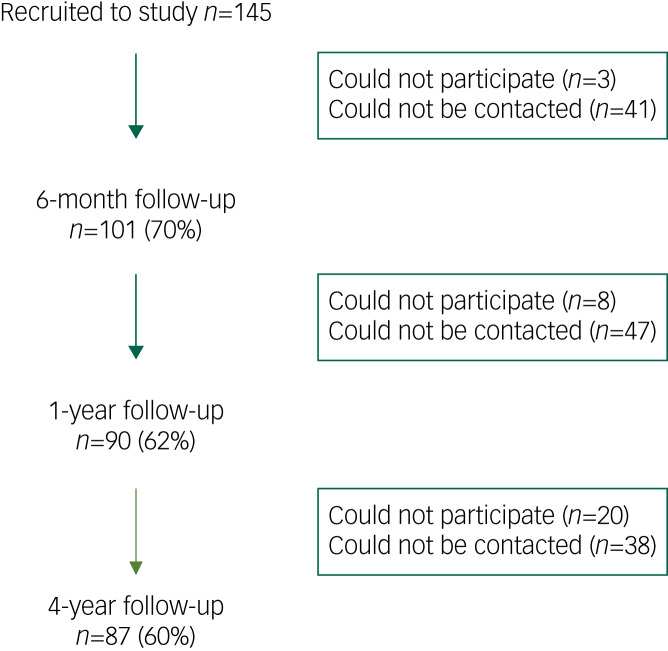
Flowchart of follow-up rates at each time point.

The study recruited 145 participants, of whom 87 were followed up at 4 years (Fig. 1). At treatment intake 71.7% of the participants were male, with a median age of 35 years; 68 (46.9%) participants reported having problem alcohol and drug use, 46 (31.7%) had problem alcohol use only and 31 (21.4%) had problem drug use only. Of the 99 participants who had problem drug use, 30 were using a single substance (with or without alcohol) and 69 were using two or more substances (with or without alcohol). Polydrug use was more common than single drug use. The most common substance of use was heroin, used by 39% of participants. Cannabis was used by 35% of participants, 29% used sedatives and 23% used methadone (illicit or prescribed) (Supplementary Table 7, available at https://dx.doi.org/10.1192/bjo.2023.23).

Study participants who did not complete the full programme but who were followed up at 1 year and 4 years (‘non-graduates’) were used as a comparison group.

### Ethical approval

The authors assert that all procedures contributing to this work comply with the ethical standards of the relevant national and institutional committees on human experimentation and with the Helsinki Declaration of 1975, as revised in 2008. All procedures involving human subjects/patients were approved by NHS Lothian Research Ethics Committee (REC ref. 08/S1101/25).

### Outcome measures

The follow-up protocol used a semi-structured interview designed to provide information about key aspects of participants’ lives. Participants were interviewed on admission and at 6-month, 1-year and 4-year follow-up stages using the ASI-X, an updated and slightly expanded version of the EuropASI.^6–8^ Sixty-six questions were used from the ASI-X. The current study analyses 7 subsets of the ASI-X (medical status, satisfaction with work, drug status, alcohol status, legal status, other social relationships and psychiatric status) recorded 4 years after leaving the programme. Further questions on interview included frequency of any alcohol use, frequency of heavy alcohol use (using the cut-off of more than 9 units per day on more than 10 days in the past 30 days, i.e. equating to more than 21 units of alcohol per week on average), frequency of heroin use, frequency of injecting drug use and abstinence status over the previous 30 days. The ASI-X subscales had acceptable to high levels of internal consistency as determined by Cronbach's alpha, as detailed by Rome et al (2017).^3^

### Procedure

Clinical staff completed an ASI-X with each participant on intake according to the training and guidance manual. A follow-up ASI-X interview, completed by telephone, was conducted at 6 months, 1 year and 4 years post-treatment by independent researchers. The independent researchers had no relationship with the treatment provider and this was made clear to participants at each contact point. Participants were contacted by telephone or mail to arrange follow-up interviews. For those participants not contactable, additional methods were used to update details and increase the frequency of contact attempts. Every out-of-contact participant was phoned morning, afternoon and evening on each day of the working week (Monday to Friday).

### Analysis

The responses were scored according to the ASI-X scoring guidance.^9^ These scores were then used to calculate composite scores for each section. The possible range of the ASI-X composite scores was between 0 and 1, where lower scores indicated more positive results.

Substance use and injecting drug use was measured as the total number of days that participants spent using each substance in the 30 days prior to the interview. Because of the use of 30-day measurement windows, the data do not provide a fully continuous coverage of the follow-up period. Abstinence rates were defined at baseline as free of substance use on admission to the programme, and at follow-up points as continuing abstinence.

Wilcoxon signed-rank tests were conducted to assess paired differences present between the various outcome measures from baseline to 4-year follow-up for the cohort as a whole, as well as for completers (‘graduate’) and non-graduate subgroups, and abstinent and non-abstinent subgroups. A correlation analysis (Spearman's rank-order correlation) was conducted to investigate stability of outcomes for individuals from the 1-year to 4-year follow-up points. Mann–Whitney *U*-tests were conducted to assess differences present between graduate and non-graduate participant groups on outcome measures at baseline and at 4-year follow-up interview. Where categorical data were analysed, χ^2^-tests were used. Further interpretation of results was conducted using descriptive statistics to illustrate the direction of any change for various outcome measures.

## Results

The cohort was found to be slightly older in median age (35 *v.* 30 years) and similar in gender (72% *v.* 71% male) to the treatment-seeking population in Scotland at the time.^10^ Approximately 55% of the participants completed the rehabilitation programme (‘graduated’). Of the 125 participants available for follow-up at 4 years, 42% identified themselves as having problematic use of both alcohol and drugs, 35% reported an alcohol only problem and 22% reported problem drug use only. Retention in treatment to the completion of the programme was highest for those in the ‘alcohol only’ group, where 65.9% graduated (compared with 50% of those reporting ‘drug only’ problem use and 49% of those who reported having problematic alcohol and drug use). At 4-year follow-up there was proportionally a greater degree of attrition from the ‘drug only’ group (Table 1). This is consistent with other research studies.^11,12^

**Table 1:** Characteristics at baseline and follow-up for those who completed treatment (‘graduates’) and those who did not (‘non-graduates’)

	Baseline (*n* = 125)		4-year (*n* = 87)		Lost to follow-up (*n* = 38)	
	Graduates	Non-graduates	Graduates	Non-graduates	Graduates	Non-graduates
All participants, *n*	69	56	56	31	13	25
Male gender, *n* (%)	48 (70)	42 (75)	39 (70)	24 (77)	9 (69)	18 (72)
Age (median), years	36	34	36.5	34	31	34
SDS score, median (IQR)	12 (4)	12 (3)	12 (5)	12 (4)	11 (2)	13 (3)
Substance use group						
Alcohol, *n*	29	15	24	10	5	5
Drugs, *n*	14	14	10	5	4	9
Alcohol and drugs, *n*	26	27	22	16	4	11

SDS, severity of dependence scale; IQR, interquartile range.

There was no significant difference in age (*U* = 1761, *P* = 0.395), Severity of Dependence Scale (SDS) scores (*U* = 2188.5, *P* = 0.199) or gender distribution (*X*^2^ = 0.453, *P* = 0.501) between the graduate group and non-graduate group. There were no significant differences between the graduate and non-graduate groups at baseline in all outcomes except the medical status score; graduates’ medical status score at baseline was significantly worse (*U* = 1487, *P* = 0.023).

Baseline measures were compared between participants followed up at 4 years and those lost to follow-up. Those followed up spent significantly longer in treatment than those not followed up (mean 63.89 *v.* 49.45 days, *U* = 1216, *P* = 0.019). There were also more graduates followed up than non-graduates (*X*^2^(1, 125) = 9.727, *P* = 0.002) (Supplementary Table 4). Those followed up were using alcohol more frequently at baseline than those not followed up (*U* = 1304.5, *P* = 0.044). There was no significant difference between the two groups on all other measures (Supplementary Table 5).

Baseline measures were compared with 4-year follow-up for all participants and then for two groups: graduates and non-graduates (Tables 2 and 3).

**Table 2:** Comparison of outcome measures from baseline to 1 year and 4 years post-treatment

Outcome measure	Baseline (*n* = 125)	1 year (*n* = 90)			4 years (*n* = 87)		
	Median (IQR)	Median (IQR)	*Z*	*P* (2-tailed)	Median (IQR)	*Z*	*P* (2-tailed)
Medical status^a^	0.168 (0.67)	0 (0.66)	−1.702	0.089	0.0 (0.72)	−0.377	0.706
Satisfaction with work^a^	0.667 (0.50)	0 (0.02)	−6.265	<0.001	0 (0.58)	−4.7762	<0.001
Alcohol status^a^	0.261 (0.60)	0 (0.18)	−4.379	<0.001	0 (0.10)	−4.600	<0.001
Drug status^a^	0.146 (0.28)	0.02 (0.12)	−4.311	<0.001	0 (0.10)	−4.440	<0.001
Legal status^a^	0 (0.35)	0 (0)	−3.405	0.001	0 (0)	−4.911	<0.001
Other relationships^a^	0.25 (0.50)	0 (0)	−6.817	<0.001	0 (0)	−5.961	<0.001
Psychiatric status^a^	0.341 (0.37)	0.41 (0.59)	−0.207	0.836	0.262 (0.61)	−0.408	0.683
Frequency of alcohol use	0 (10), mean 5.70	0 (0.5), mean 2.81	−3.425	0.001	0 (4), mean 3.79	−1.847	0.065
Frequency of heavy alcohol use	25 (12), mean 22.94	0 (0), mean 5.10	−4.581	<0.001	0 (5.5), mean 4.58	−4.012	<0.001
Frequency of heroin use	0 (4), mean 5.30	0 (0), mean 1.32	−3.157	0.002	0 (0), mean 1.09	−3.632	<0.001
Frequency of injecting drug use	0 (0), mean 2.47	0 (0), mean 0.38	−2.823	0.005	0 (0), mean 0.6	−2.753	0.006
Rates of abstinence from substances (proportion)	15/125	27/90		n = 90, *P* = 0.091	42/87		n = 87, *P* = 0.001

IQR,  interquartile range; frequency, number of days on which the stated substance was used over the previous 30 days. Means are shown where we felt they are informative. Abstinence is shown as a proportion and was analysed using a *χ*^2^-test.

a. Subscales of the Addiction Severity Index-X, composite scores range from 0–1, lower scores indicate more positive results.

**Table 3:** Comparison of outcomes for those who completed treatment (‘graduates’) with those who did not (‘non-graduates’)

	Baseline (n = 125)		4-year follow-up (n = 87)		Change score	
Outcome measure	Graduates, mean (s.d.)	Non-graduates, mean (s.d.)	Graduates, mean (s.d.)	Non-graduates, mean (s.d.)	*U*	*P*
Medical status^a^	0.376 (0.344)	0.267 (0.359)	0.327 (0.403)	0.320 (0.390)	683	0.094
Satisfaction with work^a^	0.573 (0.328)	0.591 (0.326)	0.132 (0.281)	0.462 (0.451)	596	0.016
Alcohol status^a^	0.376 (0.317)	0.268 (0.310)	0.083 (0.173)	0.194 (0.304)	626	0.013
Drug status^a^	0.134 (0.145)	0.184 (0.107)	0.049 (0.114)	0.107 (0.148)	848	0.851
Legal status^a^	0.156 (0.228)	0.180 (0.232)	0.017 (0.095)	0.049 (0.141)	811	0.569
Other relationships^a^	0.250 (0.223)	0.318 (0.079)	0.024 (0.109)	0.079 (0.204)	841	0.805
Psychiatric status^a^	0.326 (0.217)	0.346 (0.241)	0.245 (0.295)	0.402 (0.254)	562	0.007
Frequency of alcohol use	6.565 (10.02)	4.625 (8.21)	2.71 (5.284)	5.74 (8.622)	713	0.165
Frequency of heavy alcohol use	23.90 (6.14)	21.25 (8.64)	0.89 (2.47)	14.57 (10.71)	8.0	<0.001
Frequency of heroin use	4.246 (9.46)	6.607 (10.86)	0.96 (4.561)	1.32 (5.43)	778	0.330
Frequency of injecting drug use	2.522 (7.60)	2.411 (6.01)	0.55 (4.01)	0.68 (2.63)	832	0.645

Frequency, number of days on which the stated substance was used over the previous 30 days.

a. Subscales of the Addiction Severity Index-X.

### Changes associated with attendance at the rehabilitation programme

For the group as a whole, there was a significant improvement from baseline to 4 years across all but three of the outcome measures, regardless of treatment completion. There was no significant difference from baseline to 4-year follow-up in the psychiatric and medical status subscores, regardless of the substance use group participants identified with (drugs, alcohol or both). There was a non-significant trend (*P* = 0.065) towards fewer days of alcohol use at 4-year follow-up. Abstinence rates rose from 12% to 30% at 1 year and to 48% at 4 years across the group as a whole (Table 2), with a statistically significant increase in the proportion of participants reporting abstinence at 4-year follow-up compared with baseline (baseline 15/125, 4-year 42/87, *n* = 87 *P* < 0.001).

### Stability of change

Improvements seen at 1-year follow-up were maintained at 4-year follow-up, except for frequency of alcohol use, which increased at 4-year follow-up (Table 2). This comparison of 1-year data with 4-year data potentially demonstrates the maintenance of treatment effect. However, it is possible that the outcome measures at 1 year and 4 years could be identical, but that these could reflect scores for completely different subsets of individuals. For this reason, a correlation analysis (Spearman's rank-order correlation) was conducted to look at stability of outcomes for individuals. There was statistically significant moderate positive correlation between medical status scores (*r_s_*(65) = 0.400, *P* = 0.001), alcohol status (*r_s_*(65) = 0.253, *P* = 0.038), drugs status (*r_s_*(65) = 0.395, *P* = 0.001), legal status (*r_s_*(79) = 0.357, *P* = 0.001), psychiatric status (*r_s_*(65) = 0.529, *P* > 0.001), alcohol use (*r_s_*(65) = 0.380, *P* = 0.002) and heroin use (*r_s_*(65) = 0.396, *P* = 0.001). There was a positive correlation of moderate strength between these 1-year and 4-year subscores, which could be consistent with the view that there is stability of change over time.

There was no significant correlation between satisfaction with work scores (*r_s_*(65) = 0.219, *P* = 0.075), other relationships status scores (*r_s_*(65) = −0.060, *P* = 0.631) or frequency of injecting drug use (*r_s_*(65) = 0.161, *P* = 0.193) at 1 year and at 4 years. Closer inspection of the data revealed that although half of the participants improved their work satisfaction score from 1 year to 4 years, half worsened. There was further improvement in the other relationships subscores from 1 year to 4 years, although the change did not reach a level of significance. The sample size for injecting drug users was small and may lack statistical power.

### Changes associated with programme completion

**Fig. 2:**
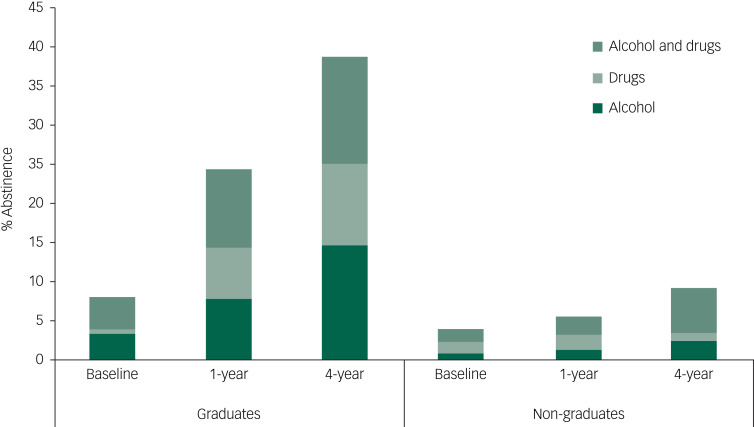
Abstinence rates at follow-up points for those who completed treatment (‘graduates’) and those who did not (‘non-graduates’).

Graduates showed greater positive change in scores than non-graduates at 4 years for five outcome measures; work satisfaction, alcohol status, psychiatric status, heavy alcohol use and abstinence rates (Table 3). Graduates’ scores improved more than non-graduates’ scores for work satisfaction, alcohol status subscores and frequency of heavy alcohol use. The graduate group's psychiatric status score improved at 4-year follow-up, whereas the non-graduate group's score worsened. Both groups showed improved drug status, other relationship status, legal status, heroin use and injecting drug use outcome measure scores, with no significant difference in change scores. There was no significant change in medical status scores for either group. Both groups showed improved abstinence rates (Fig. 2), but the improvement was greater in the graduate group (χ^2^(2, 87) = 9.738, *P* = 0.002). The graduate group's abstinence rates rose from 14.5% (10/69) at baseline to 60.7% (34/56), compared with a change from 8.9% (5/56) to 25.8% (8/31) in the non-graduate group. When analysing results by substance use group, the results were significant for each of the three groups (the alcohol group's abstinence rate increased from 5/44 to 15/34, *n* = 34, *P* = 0.002; drugs only group increased from 3/28 to 10/15, *n* = 15, *P* = 0.012; alcohol and drugs group increased from 7/53 to 17/38, *n* = 38, *P* = 0.002).

### Changes associated with abstinence

Baseline to 4-year outcomes were compared separately for those who reported being abstinent from all substances at 4 years and those who did not, and the change scores for the two groups were then compared (Supplementary Table 6).

For the group who were abstinent at 4 years, all ASI-X status outcome measures improved from baseline to follow-up. For the group who were not abstinent at 4 years, medical and psychiatric status scores worsened significantly, and there was no significant difference in the alcohol status composite score from baseline to follow-up. The change scores between the two groups differed significantly for medical status, alcohol status and psychiatric status. Remaining abstinent from substances at follow-up was associated with better outcomes in the medical, psychiatric and alcohol subsections.

### Mortality

There were 12 deaths within the cohort over the 4-year follow-up period. Two of these deaths occurred 6–12 months after leaving the rehabilitation programme, two deaths occurred 1–2 years after, 5 occurred 2–3 years after and the remainder occurred over 3 years after leaving the programme. This mortality rate of 20.7 per 1000 people is approximately 5 times higher than that of the general population of Scotland under 75 years old in 2014.^13^ However, this rate is commensurate with mortality rates found in other cohort studies of drug users.^14,15^ Five of these deaths were classed as ‘drug-related’ in line with the definition of the Advisory Council on the Misuse of Drugs. The rate of drug-related deaths of 8.6 per 1000 is commensurate with national rates of drug-related deaths and local rates of drug-related deaths of 8.8 per 1000 problem drug users within Lothian Health Board.^16^ Alcohol-related health complications accounted for one-third of the deaths, and 25% of the deaths were secondary to respiratory conditions. Of the 12 participants who died, 8 had reported using both alcohol and drugs, 2 had reported only problem alcohol use and 2 had reported problem drug use only. Four of the participants who died had not completed LEAP, whereas the other eight had. Baseline outcome scores were not significantly different for these 12 participants in severity of dependence score, frequency of use of alcohol or heroin or ASI-X subsection scores. A high burden of physical illness in those admitted to in-patient substance misuse services has been described in the literature.^14,17,18^

## Discussion

### Statement of principle finding

Attending the LEAP abstinence-based rehabilitation programme was associated with positive changes in psychological and social well-being and harm reduction from substance use at 4-year follow-up, demonstrating stability of change from 1-year to 4-year follow-up, regardless of whether the programme was completed or not. However, completing the full 12-week programme (55% of participants completed) was associated with drinking less alcohol and drinking less frequently, and with improvements in alcohol status score, work satisfaction score and psychiatric status score on the ASI-X, in comparison with non-completion. Abstinence rates improved from 12% at baseline to 30% at 1 year and 48% at 4 years. Similar abstinence rates at 1-year follow-up after residential rehabilitation were recorded by Gossop et al, who reported 33% at 1 year and 2 years, and 38% at 5 years.^19^ This improvement in abstinence was most marked in those who graduated the programme, where rates rose from 14.5% to 60.7%. Further analysis of those who reported being abstinent from all substances at 4 years found significant improvement in medical, psychiatric and alcohol subsections, compared with those who were not abstinent. These results add to the growing evidence that positive changes in behaviour after residential rehabilitation for substance misuse can be maintained over time.

### Relevance to other studies

Treatment retention, completion and continuing care post-discharge have been found to be significant predictors of recovery.^3,20–28^

The association between programme completion and better outcomes demonstrated by our study has been found in previous larger-scale longitudinal cohort studies. Gossop et al, during a large multi-site cohort study of a variety of modes of treatment in England, reported that completion of both residential rehabilitation and in-patient detoxification programmes was predictive of positive outcomes and associated with a reduced risk of relapse.^29^ Teesson et al reported on a longitudinal Australian study that found more time spent in residential rehabilitation and successful completion of treatment were associated with reduced drug use and criminal behaviour and increased abstinence.^25^ Graduation from the treatment programme was of greater importance for sustained abstinence than programme type or length. This finding was supported by another Australian study, which found that significant predictors of treatment success were completion of primary index treatment and attendance at mutual aid. Further, for individuals with primarily problem alcohol use, abstinence was predicted by residential rehabilitation and mutual aid attendance.^26^ Hubbard et al in the USA reported that participants who spent 90 days or more in long-term residential treatment had the most significant reductions in drug use and criminal activity and significant increases in rates of employment.^27^ Further research has been conducted on factors affecting retention in treatment. Meier & Best found that retention rates for 90 days of residential treatment in the UK varied from 25 to 48%.^30^ Programme-level factors associated with retention were primarily related to patient privacy, higher staff/patient ratio and domestic services support and individual counselling, with higher levels linked with greater patient retention. Greater programme intensity and higher numbers of beds per facility were both associated with lower retention rates for 90 days.

Our finding that the graduate group achieved and maintained abstinence to a greater degree than the non-graduates, and that abstinence was associated with improved outcomes, is also consistent with previous work. Gossop et al reported abstinence rates of 35.9% for participants in contact with residential rehabilitation services and 24.3% for those receiving methadone maintenance treatment at 2-year follow-up.^24,29^ They found better rates of abstinence with treatments that have a clear abstinence focus, rather than a focus on harm reduction. A longitudinal cohort study of treatment outcomes associated with a range of treatment agencies in Scotland reported abstinence rates of 8% at 33-month follow-up, but when assessed according to mode of treatment this rate was 24.7% for those treated in residential rehabilitation services.^31^ In a comparison between abstinent and non-abstinent participants, abstinence was associated with reduced criminal behaviour, suicide/self-harm and alcohol misuse, and with improvements in employment and physical health. Teesson et al reported that heroin abstinence was more likely in a group who had engaged in residential rehabilitation, who also demonstrated lower criminality and lower likelihood of needle-sharing.^25^ Manning et al compared the effectiveness of linkage to 12-step mutual aid groups for 153 participants from a short-term in-patient detoxification. Assertive linkage to 12-step mutual aid groups led to more frequent attendance post-discharge, which was associated with improved abstinence.^32^ The importance of peer support and assertive linkage was underlined, and this adds to the literature on continuity of care.

The current findings that the frequency of alcohol use and quantity of alcohol consumed were reduced is important in the context of physical and psychological harm that result from problem alcohol use. Chronic alcohol misuse is an important cause of medical complications among drug misusers and has been found to be associated with increased risk of overdose and mortality.^33^ In the present study, 77% of participants identified themselves as having problem alcohol use, either alone or in combination with problem drug use. However, although frequency of alcohol use improved from baseline to 1-year follow-up, this was not maintained at 4 years, when there was an increase in use of alcohol (albeit still lower than at baseline). This has implications for clinicians and policymakers; excessive alcohol use and public health policies regarding alcohol may warrant a renewed focus.

Psychological and psychiatric problems are more prevalent among problem drug and alcohol users in treatment than in the general population. Improvement in psychological health and functioning is an important treatment goal in substance misuse. The current finding that positive improvements were observed in the psychiatric subscore of LEAP graduates, compared with a worsening score in non-graduates, suggests that gains in mental health can be achieved after completing residential treatment. It may be that the LEAP service is better placed and equipped to assess and address the psychiatric needs of their patients, in comparison with other treatment models (such as short-term detoxification treatments or methadone maintenance treatment).^34,35^ Additionally, participants who reported that they were abstinent at 4-year follow-up had significantly improved psychiatric and medical status scores, indicating gains are more likely in physical and mental health if abstinence is maintained. This has important service development implications, highlighting the importance of thorough psychiatric assessment in routine clinical practice and the positive impact of retaining individuals in treatment. A failure to adequately address psychiatric problems leads to poorer outcomes from substance misuse treatment.^14,29^ This implies the importance of enhanced training for staff in both psychiatric settings and substance misuse services, and the importance of strong links between mental health services and substance misuse services.

### Strengths and weaknesses of the study

The findings outlined above should be interpreted cautiously. This kind of naturalistic longitudinal cohort study design does not permit changes observed to be attributed directly to the impact of residential treatment. We have identified an association between outcome measures and the intervention, rather than a causal explanation. Residential rehabilitation typically forms one of multiple interactions with substance use treatment services, and delineating which of these treatments has produced specific outcomes in the longer-term is therefore challenging. However, studies of this design allow investigation of treatment outcomes under normal clinical practice conditions and are valuable in helping to identify what works in practice, even though control over aspects of evaluation design are more limited than in other experimental studies. Regardless of whether the results are directly attributable to the treatment programme, the outcomes reflect the participants’ recovery progress over 4 years. The discovery that outcomes were better for those who completed the residential programme compared with those who did not would support the conclusion that at least some of the positive outcomes at 4 years is attributable to the intervention.

The assessment of change over time post-treatment can be affected by many factors. Participants may experience change, either positive or negative, in aspects of their lives that leads them to decide not to participate further, and people with drug and alcohol problems are often socially and geographically transient and lose touch with researchers. Several studies provide good evidence that tracking patient outcomes over time post-discharge through use of validated assessment measures can provide important insights into recovery trajectories.^21,35,36^ However, attrition from research follow-ups was a major methodological limitation for many studies. A notable strength of this study was the high baseline recruitment of 100% of eligible patients and good retention rates over an extended period of time (60% of the original cohort and 70% of those available for follow-up at 4 years). Analysis showed that there were no pre-treatment differences between those who were followed up and those who were not. Those successfully interviewed at 4 years had spent more time in rehab and a greater proportion had completed treatment. The improvements we have obtained may be from a group of individuals who were better placed to maintain their lifestyle changes compared with those not followed up. Higher rates of assessment data at all collection time points would have increased the generalisability of study results and limited any potential systematic bias (the potential that those who were followed up differed systematically from those who were not).

The use of 30-day measurement windows and relying on self-reported levels of substance use mean that the data do not provide a fully continuous coverage of the follow-up period and are reliant on accurate self-reporting. Neale & Robertson found a high level of consistency between self-reported drug use and oral fluid testing, in fact discovering that respondents commonly reported consumption that screening failed to identify.^37^ The use of the extensive, validated and reliable ASI-X outcome measure allows the accurate study of a range of domains. A growing number of studies have used the ASI to measure change in substance use, which improves comparability of outcomes across studies and enables the examination of treatment effects on a range of life domains.^38–41^

### Future directions

Ongoing research is required to further delineate the mechanisms by which residential rehabilitation generates outcomes and the ways in which these differ from other treatment modalities. Given the greater change associated with retention in treatment, it would be important to understand what factors are associated with treatment completion and to design residential services to maximise retention in treatment. Understanding the factors that promote abstinence will allow more focused interventions and support and lead to improved long-term outcomes for problem substance users. Residential rehabilitation services can be uniquely placed to offer increased support for physical and mental health issues, to help develop a supportive network of peers and to encourage integration into a wider treatment network. Future research could explore the effect of strong links between community services and rehabilitation programmes, supporting patients before, during and after rehabilitation, on the stability of recovery over time.

Our findings provide an encouraging view of residential rehabilitation programmes. Wherever an individual lives in Scotland they should be able to access an array of substance use services, both harm reduction and abstinence focused, at the point of need.

## Data Availability

The data that support the findings of this study are available from the corresponding author on reasonable request. The data are not publicly available because they contain information that could compromise the privacy of research participants.

## Supplementary material

For supplementary material accompanying this paper visit http://doi.org/10.1192/bjo.2023.23. click here to view supplementary material
