# Supplementary material for: Substance use, risk behaviours and well-being after admission to a quasi-residential abstinence-based rehabilitation programme: 4-year follow-up
Source: BJPsych Open. 2023 Mar 13;9(2):e52. doi: 10.1192/bjo.2023.23 (PMC10043999; doi:10.1192/bjo.2023.23)
Supplement: Supplementary file 1 [file S2056472423000236sup001.docx]

Supplementary Materials

Tables 4, 5, 6 & 7:

| **Variable** | **Followed up n=87** | **Not followed up n=38** | **Statistical Analysis** |
| --- | --- | --- | --- |
| Age | Mean 37.4, Median 35,  Range 21-60 | Mean 34.92, Median 33.5, Range 21-56 | U=1395, *p* = .166 |
| Gender | M 63 (72.4%)  F 24 (27.6%) | M 27 (71.1%)  F 11 (28.9%) | X^(1)^ = 0.024 (df1)  *p* = .876 |
| SDS scores | Median 12  Range 0-24 | Median 12  Range 0-15 | U=1491.5, *p* = .382 |
| Substance use group | A 34 (39.1%)  D 15 (17.2%)  AD 38 (43.7%) | A 10 (26.3%)  D 13 (34.2%)  AD 15 (39.5%) | X^(1)^ = 4.734 (df2)  *P* = .095 |
| Graduate status | G 56 (64.4%)  NG 31 (35.6%) | G 13 (34.2%)  NG 25 (65.8%) | X^(1)^ = 9.727 (df1)  *P* = .002 |
| Days in treatment | Median 79  IQR 48 | Median 51.5  IQR 61 | U=1216  *P* = .019 |
| Abstinent at baseline | Abstinent: 8 (9.2%)  Not A: 79 (90.8%) | Abstinent: 7 (18.4%)  Not A: 31 (81.6%) | X^(1)^ = 2.132 (df1)  *p* = .144 |

**Table 4**: Attrition analysis; demographics, time in treatment.

*Notes: SDS=severity of dependence score, A=alcohol only, D=drugs only, AD=alcohol & drugs, G=graduate, NG=non-graduate, IQR=interquartile range*

| **Substance** | **% subjects using substance** | **Mean no. of days of use (SD)** | **Range** | **IQR** | **Median** |
| --- | --- | --- | --- | --- | --- |
| alcohol | 79 | 8.2 (SD10.79) | 0-30 | 15.0 | 2.0 |
| heroin | 39 | 13.6 (SD 12.16) | 0-30 | 27.0 | 8.0 |
| cannabis | 35 | 12.2 (10.62) | 1 30 | 18.0 | 9.0 |
| sedatives | 29 | 14.1 (SD 12.63) | 1 30 | 27.0 | 8.5 |
| methadone | 23 | 24.6 (SD 9.19) | 1 30 | 10.0 | 30.0 |
| other opiates | 15 | 14.4 (SD 12.40) | 0-30 | 28.0 | 14.0 |
| cocaine | 11 | 5.1 (SD 6.82) | 1 28 | 2.25 | 2.5 |
| amphetamines/ hallucinogens /inhalants/ other | 6 | 16.1 (SD 12.11) | 3 30 | 23.25 | 12.5 |

**Table 7**: Substances of use by percentage of subjects. Frequency is the mean number of days of use in the past 30 days

*Notes: SD=standard deviation, IQR=interquartile range*
